# Supplementary material for: Chasing collective variables using temporal data-driven strategies
Source: QRB Discov. 2023 Jan 6;4:e2. doi: 10.1017/qrd.2022.23 (PMC10411323; doi:10.1017/qrd.2022.23)
Supplement: Supplementary file 1 [file S2633289222000230sup001.pdf]

QRB Discovery (2022), 1–6
doi:10.1017/qrd.2022.xx

Supplementary materials

CAMBRIDGE
UNIVERSITY PRESS

Chasing collective variables using temporal data-driven strategies

Haochuan Chen[1] and Christophe Chipot[1,2,3*]

[1]Laboratoire International Associé Centre National de la Recherche Scientifique et University of Illinois at Urbana-Champaign, Unité Mixte de Recherche n°7019, Université de Lorraine, B.P. 70239, 54506 Vandœuvre-lès-Nancy cedex, France, [2]Theoretical and Computational Biophysics Group, Beckman Institute, and Department of Physics, University of Illinois at Urbana-Champaign, Urbana, Illinois 61801, USA, [3]Department of Biochemistry and Molecular Biology, University of Chicago, Chicago, Illinois 60637, USA
*Corresponding author. E-mail: chipot@illinois.edu

(Received xx xxx xxxx)

Guidelines for choosing the neural-network hyperparameters

The Siamese neural-network (NN) models presented in this contribution should fulfill two tasks, namely (i) capture the nonlinearity of the input features, and (ii) reduce the dimensionality of the process at hand. The first task requires more layers and neurons for the middle layers, while the second task suggests that the number of neurons should be decreased from the first layer to the last one, as shown in the original VAMPnets (Mardt et al., 2018). To strike a balance between task (i) and the computational cost of the overall optimization, while avoiding potential overfitting, the size of the NN, or the number of layers and neurons, cannot be blindly increased. In addition, the number of neurons in the first, or input layer is determined by the number of candidate CVs. The number of neurons in the last layer should match the number of eigenfunctions in the state-free reversible VAMPnets (SRVs). With all these requirements in mind, the guidelines for configuring the layers and neurons are: (a) relate the number of neurons of the first layer to the number of candidate CVs, (b) use two- or three-fold neurons in the second layer, (c) ensure that the number of neurons in the last layer is the same as the number of desired eigenfunctions, and (d) insert intermediate layers between the second and the last layers with decreasing number of neurons.

When the biasing force F_{bias}, is applied on the learned CV, $\xi(\mathbf{s})$, it is propagated to the input features $\mathbf{s}$ using the chain rule through $F_{\text{bias}} \cdot \nabla_{\mathbf{s}}\xi(\mathbf{s})$, which requires $\xi(\mathbf{s})$ to have a continuous derivative with respect to $\mathbf{s}$, which in turn, implies that the activation functions in $\xi(\mathbf{s})$ should also have continuous derivatives with respect to their input values. Bearing this requirement in mind, in the NANMA and trialanine test cases, we have, respectively, selected the hyperbolic tangent (tanh) and the exponential linear unit (ELU) as activation functions for all layers.

© The Authors(s) 2022. This is an Open Access article, distributed under the terms of the Creative Commons Attribution licence (http://creativecommons.org/licenses/by/4.0/), which permits unrestricted re-use, distribution, and reproduction in any medium, provided the original work is properly cited.

Discretization issue of the space of the learned CVs in the trialanine case

As a variant of the adaptive biasing force (ABF) method (Darve and Pohorille, 2001; Comer et al., 2015), the well-tempered meta-extended ABF (WTM-eABF) (Fu et al., 2019) relies on discretizing the CV space to be sampled by multidimensional grids of finite bins, and building histograms to compute the free-energy gradients. In general, if more bins are used for a given grid, the potential of mean force (PMF) integrated from the free-energy gradients is expected to be more accurate, assuming the calculation is converged, but it would also take a longer time to achieve an acceptably uniform sampling. Conversely, if less bins are used, then the accuracy of the PMF is likely to diminish. In addition, in WTM-eABF, the PMF could be also affected by the spring force constants utilized in the extended Lagrangian dynamics, which are equal to $k_{\mathrm{B}}T/\sigma^2$, where σ is commonly chosen as the width of the bins (Fu et al., 2016).

The blue and red pathways in Figure 4H are, respectively, determined from the three-dimensional free-energy landscapes along the learned CVs (ξ_1, ξ_2, ξ_3), on the one hand, and along the known reference CVs (ϕ_1, ϕ_2, ϕ_3), on the other hand. All ϕ angle range from -180° to +180°, and they are discretized in narrow bins of width equal to 5°, guided by physical intuition, as isomerization is expected to be a gradual process involving sequential changes in the dihedral angles. As a result, there are 72×72×72=373,248 bins for (ϕ_1, ϕ_2, ϕ_3). As for the NN-learned (ξ_1, ξ_2, ξ_3), we evidently cannot rely on our intuition, and must consequently discretize the learned conformational space into as many bins as possible (150×150×150 bins), which, for a fixed simulation time–equal to that necessary to achieve uniform sampling in the (ϕ_1, ϕ_2, ϕ_3) space, might lead to a relatively poor convergence, while requiring different spring force constants for WTM-eABF. The likely suboptimal convergence of our simulation can explain the slight deviation of the barriers between the two pathways (blue and red in Figure 4H), although both of them visit A-M_1-M_3-B. It should, however, be emphasized that after reweighting the trajectory from (ξ_1, ξ_2, ξ_3) to (ϕ_1, ϕ_2, ϕ_3) in 72×72×72 bins, as shown in the green curve in Figure 4H, the barriers are quantitively close to the reference (red).

Parameters and simulation details of the iterative learning in the NANMA and trialanine cases

Tensorflow (Abadi et al., 2015) and NAMD (Phillips et al., 2020) with Colvars (Fiorin et al., 2013; Chen et al., 2022) were used for training the neural networks and running simulations, respectively. The parameters of the iterative learning, including the candidate CVs, time lags, network structures, normalization factors, optimization, number of iterations, number of CVs used in each iteration, number of CVs for the final free-energy calculations, simulation time during the different iterations, simulation time of the final free-energy calculations, simulation timesteps, force fields, output frequencies of the trajectories, and other parameters of WTM-eABF simulations used can be found in Table 1. The Adam optimizer (Kingma and Ba, 2015) was used for all trainings. The datasets were splitted into training sets and validation sets by a ratio of 9:1, and the trainings were stopped if the losses of validation sets were not decreased further in 20 epochs (early stopping). The boundaries of the learned CVs were

determined by transforming the training datasets into the values of the learned CVs, finding the minima and maxima, and then extending the ranges by a factor of 10%. More specifically, the lower and upper boundaries are $\xi_{\mathrm{min}} - 0.05(\xi_{\mathrm{max}} - \xi_{\mathrm{min}})$ and $\xi_{\mathrm{max}} + 0.05(\xi_{\mathrm{max}} - \xi_{\mathrm{min}})$, respectively. The spring force constants of WTM-eABF simulations were determined by $k_{\mathrm{B}}T/\sigma^2$, where the σ was calculated the same as the widths of bins.

Table 1. Paramters of iterative learning of the NANMA and the trialanine cases

Type of parameters	Parameter	NANMA	Trialanine
Neural network	Candidate CVs (input features)	$\sin(\phi), \sin(\psi)$, $\cos(\phi), \cos(\psi)$	$\sin(\phi_1), \sin(\phi_2), \sin(\phi_3)$, $\sin(\psi_1), \sin(\psi_2), \sin(\psi_3)$, $\cos(\phi_1), \cos(\phi_2), \cos(\phi_3)$, $\cos(\psi_1), \cos(\psi_2), \cos(\psi_3)$
	Time lag	0.025 ps	0.25 ps
	Network structure	4-12-10-8-6-4-2	12-32-24-16-8-3
	Activation functions	linear-tanh-tanh-tanh-tanh-tanh-linear	linear-tanh-elu-elu-elu-linear
	L2 normalization factors	0.0005 for all layers	0.001 for all layers
	Batch size	22,550	10,950
	Epochs	2,000	1,000
	Number of iterations	10	10
	Number of CVs used in iterations	1	2
MD simulation	Number of CVs used for the final PMF calculation	1	3
	Simulation time during iterations	100 ns	160 ns
	Simulation time of the last iteration	300 ns	310 ns
	Timestep	0.5 fs	0.5 fs
	Temperature	300 K	300 K
	Force field	CHARMM22 (MacKerell et al., 1998)	AMBER ff14SB (Maier et al., 2015)
	Output frequency of trajectories	Every 10 steps	Every 50 steps
Free enegy calculation by WTM-eABF	Number of bins used in PMF calculations during iterative training	220	48,400 (220×220)
	Number of bins of the final PMF calculations	220	3,375,000 (150×150×150)
	Height of initial the Gaussian hill of metadynamics	0.1 kcal/mol	0.1 kcal/mol
	Standard deviation of Gaussian hills	4 × bin widths for each CV	4 × bin widths for each CV
	Frequency of depositing new Gaussian hills	Every 1000 steps	Every 1000 steps

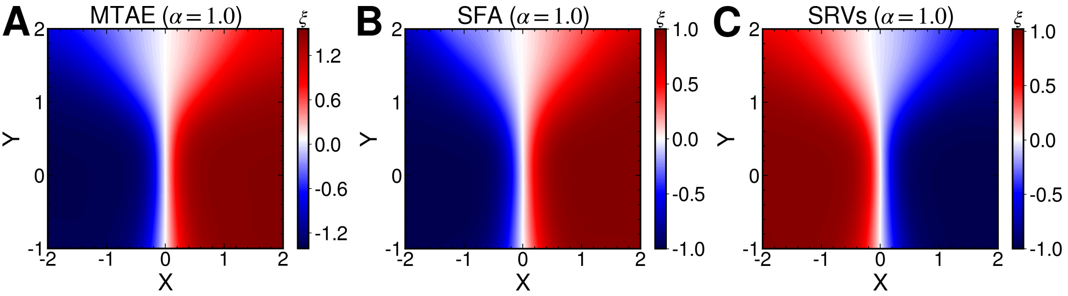

Figure 1. Projections of the one-dimensional learned CV ξ from an unbiased trajectory when $\alpha = 1.0$ by modified TAE (A), SFA (B) and SRVs (C). The learned CV in all three methods evolves mainly along the x-axis.

References

Abadi, M., Agarwal, A., Barham, P., Brevdo, E., Chen, Z., Citro, C., Corrado, G. S., Davis, A., Dean, J., Devin, M., Ghemawat, S., Goodfellow, I., Harp, A., Irving, G., Isard, M., Jia, Y., Jozefowicz, R., Kaiser, L., Kudlur, M., Levenberg, J., Mané, D., Monga, R., Moore, S., Murray, D., Olah, C., Schuster, M., Shlens, J., Steiner, B., Sutskever, I., Talwar, K., Tucker, P., Vanhoucke, V., Vasudevan, V., Viégas, F., Vinyals, O., Warden, P., Wattenberg, M., Wicke, M., Yu, Y., and Zheng, X. (2015). TensorFlow: Large-scale machine learning on heterogeneous systems. Software available from tensorflow.org.

Chen, H., Liu, H., Feng, H., Fu, H., Cai, W., Shao, X., and Chipot, C. (2022). MLCV: Bridging machine-learning-based dimensionality reduction and free-energy calculation. *Journal of Chemical Information and Modeling*, 62(1):1–8.

Comer, J., Gumbart, J. C., Hénin, J., Lelièvre, T., Pohorille, A., and Chipot, C. (2015). The adaptive biasing force method: Everything you always wwanted to know but were afraid to ask. *The Journal of Physical Chemistry B*, 119(3):1129–1151.

Darve, E. and Pohorille, A. (2001). Calculating free energies using average force. *The Journal of Chemical Physics*, 115(20):9169–9183.

Fiorin, G., Klein, M. L., and Hénin, J. (2013). Using collective variables to drive molecular dynamics simulations. *Molecular Physics*, 111(22-23):3345–3362.

Fu, H., Shao, X., Cai, W., and Chipot, C. (2019). Taming rugged free-energy landscapes using an average force. *Acc. Chem. Res.*, 52:3254–3264.

Fu, H., Shao, X., Chipot, C., and Cai, W. (2016). Extended adaptive biasing force algorithm. An on-the-fly implementation for accurate free-energy calculations. *Journal of Chemical Theory and Computation*, 12(8):3506–3513.

Kingma, D. P. and Ba, J. (2015). Adam: A Method for Stochastic Optimization. In Bengio, Y. and LeCun, Y., editors, *3rd International Conference on Learning Representations, ICLR 2015, San Diego, CA, USA, May 7-9, 2015, Conference Track Proceedings*.

MacKerell, A. D., Bashford, D., Bellott, M., Dunbrack, R. L., Evanseck, J. D., Field, M. J., Fischer, S., Gao, J., Guo, H., Ha, S., Joseph-McCarthy, D., Kuchnir, L., Kuczera, K., Lau, F. T. K., Mattos, C., Michnick, S., Ngo, T., Nguyen, D. T., Prodhom, B., Reiher, W. E., Roux, B., Schlenkrich, M., Smith, J. C., Stote, R., Straub, J., Watanabe, M., Wiórkiewicz-Kuczera, J., Yin, D., and Karplus, M. (1998). All-atom empirical potential for molecular modeling and dynamics studies of proteins. *The Journal of Physical Chemistry B*, 102(18):3586–3616.

Maier, J. A., Martinez, C., Kasavajhala, K., Wickstrom, L., Hauser, K. E., and Simmerling, C. (2015). ff14sb: Improving the accuracy of protein side chain and backbone parameters from ff99sb. *Journal of Chemical Theory and Computation*, 11(8):3696–3713.

Mardt, A., Pasquali, L., Wu, H., and Noé, F. (2018). VAMPnets for deep learning of molecular kinetics. *Nature Communications*, 9(1):5.

Phillips, J. C., Hardy, D. J., Maia, J. D. C., Stone, J. E., Ribeiro, J. V., Bernardi, R. C., Buch, R., Fiorin, G., Hénin, J., Jiang, W., McGreevy, R., Melo, M. C. R., Radak, B. K., Skeel, R. D., Singharoy, A., Wang, Y., Roux, B., Aksimentiev, A., Luthey-Schulten, Z., Kalé, L. V., Schulten, K., Chipot, C., and Tajkhorshid, E. (2020). Scalable molecular dynamics on CPU and GPU architectures with NAMD. *The Journal of Chemical Physics*, 153(4):044130.
